# Association of common *TMPRSS6 and TF* gene variants with hepcidin and iron status in healthy rural Gambians

**DOI:** 10.1038/s41598-021-87565-5

**Published:** 2021-04-13

**Authors:** Momodou W. Jallow, Susana Campino, Andrew M. Prentice, Carla Cerami

**Affiliations:** 1 Nutrition Theme, MRC Unit, The Gambia at London School of Hygiene & Tropical Medicine, Atlantic Boulevard, Fajara, P.O. Box 273, Banjul, The Gambia; 2 Department of Infection Biology, Faculty of Infectious and Tropical Diseases, London School of Hygiene & Tropical Medicine, Keppel Street, London, WC1E 7HT UK

**Keywords:** Genotype, Genetic predisposition to disease, Genetics, Biomarkers

## Abstract

Genome-wide association studies in Europeans and Asians have identified numerous variants in the transmembrane protease serine 6 (*TMPRSS6*) and transferrin (*TF*) genes that are associated with changes in iron status. We sought to investigate the effects of common *TMPRSS6* and *TF* gene SNPs on iron status indicators in a cohort of healthy Africans from rural Gambia. We measured iron biomarkers and haematology traits on individuals participating in the Keneba Biobank with genotype data on *TMPRSS6* (rs2235321, rs855791, rs4820268, rs2235324, rs2413450 and rs5756506) and *TF* (rs3811647 and rs1799852), n = 1316. After controlling for inflammation, age and sex, we analysed the effects of carrying either single or multiple iron-lowering alleles on iron status. *TMPRSS6* rs2235321 significantly affected plasma hepcidin concentrations (AA genotypes having lower hepcidin levels; F ratio 3.7, P = 0.014) with greater impact in individuals with low haemoglobin or ferritin. No other *TMPRSS6* variant affected hepcidin. None of the *TMPRSS6* variants nor a *TMPRSS6* allele risk score affected other iron biomarkers or haematological traits. *TF* rs3811647 AA carriers had 21% higher transferrin (F ratio 16.0, P < 0.0001), 24% higher unsaturated iron-binding capacity (F ratio 12.8, P < 0.0001) and 25% lower transferrin saturation (F ratio 4.3, P < 0.0001) compared to GG carriers. *TF* rs3811647 was strongly associated with transferrin, unsaturated iron-binding capacity (UIBC) and transferrin saturation (TSAT) with a single allele effect of 8–12%. There was no association between either *TF* SNP and any haematological traits or iron biomarkers. We identified meaningful associations between *TMPRSS6* rs2235321 and hepcidin and replicated the previous findings on the effects of *TF* rs3811647 on transferrin and iron binding capacity. However, the effects are subtle and contribute little to population variance. Further genetic and functional studies, including polymorphisms frequent in Africa populations, are needed to identify markers for genetically stratified approaches to prevention or treatment of iron deficiency anaemia.

## Introduction

The discovery of hepcidin and the molecular mechanisms modulating its relationship with iron metabolism have brought new insights into how iron is regulated in the human body^1,2^. Subsequently, several genome-wide studies (GWASs) have revealed single nucleotide polymorphism (SNPs) in genes involved in hepcidin regulatory pathways, that are associated with impaired iron status^3–5^. The most common SNPs associated with low iron status are in the *TMPRSS6* gene, encoding the matriptase-2 protein^6–8^. *TMPRSS6* suppresses hepcidin synthesis, and its impaired function has been associated with inappropriately high hepcidin, which restricts iron absorption by the duodenum and iron mobilisation from storage^6,9,10^. Impaired *TMPRSS6* activity has been implicated in the development of iron-refractory iron deficiency anaemia (IRIDA)^8^.

So far, more than 50 SNPs within the *TMPRSS6* gene have been reported to be associated with impaired iron status. The most commonly reported SNPs are rs855791 and rs4820268 and rs2235321^3,4,11–14^. However, most studies linking *TMPRSS6* SNPs and low iron status were conducted in Europeans and Asians. Genetic variations in the *TMPRSS6* gene has been linked to variations in iron status indicators in different populations across the world, including in India^12^, Turkey^15^ and Australia^16^.

Also, SNPs in the transferrin (*TF*) gene have been associated with altered iron status^17–19^. The most common *TF* SNP to be associated with the risk of iron deficiency is rs3811647^17,20–22^. This SNP is associated with low iron status in different populations globally, including in African populations^23^. However, little information exists on the effect *TF* SNPs on low iron status, particularly in settings with high anaemia burden.

Despite efforts to identify genetic risk factors for anaemia, very few such studies have been reported from sub-Saharan Africa^24^. To the best of our knowledge, no study has been done in West Africa to assess the effects of genetic variants in hepcidin and iron regulatory genes on low iron status. This is particularly important given that West Africa is one of the regions with the highest prevalence of anaemia^25^. In this study, we investigated the association between common SNPs in the *TMPRSS6* and *TF* genes, and iron indicators in healthy individuals from the rural Gambia.

## Results

Baseline characteristics of the study population are presented in Table 1. Due mostly to out-migration of males there was a slight sex bias (54.2% were female). There were significant differences between the sexes in age, RBC count and RBC indices, serum iron and transferrin.

**Table 1:** Demographic characteristics of the study population.

Variables	All (n = 1316)	Males (n = 595)	Female (n = 721)	*P*-value
Age, median (range)	9 (1, 87)	11.5 (1, 79)	19.7 (1, 87)	0.000
Hb (g/dl)	11.6 (6.5, 16.0)	11.6 (8.2, 16.0)	11.6 (6.5, 15.3)	0.056
RBC (× 10^12^)	4.20 (2.41, 5.62)	4.30 (2.90, 5.61)	4.21 (2.41, 5.65)	0.000
MCV (fl)	78.9 (51.3, 103.2)	78.2 (51.3, 103.2)	79.6 (52.7, 97.9)	0.000
Haematocrit (%)	33.0 (20.1, 48.10)	32.9 (24.3, 48.1)	33.1 (20.1, 44.9)	0.374
RDW (%)	14.6 (12.7, 27.4)	14.6 (12.8, 24.0)	14.5 (12.7, 27.4)	0.320
MCH (pg)	27.6 (16.2, 34.9)	27.4 (16.2, 34.2)	27.9 (17.3, 34.9)	0.005
MCHC (g/dl)	34.9 (28.9, 37.2)	35.1 (29.9, 37.2)	34.8 (28.9, 37.1)	0.024
Serum iron (umol/l)	12.15 (0.60, 52.40)	11.85 (1.7, 28.9)	12.50 (0.6, 52.4)	0.005
Hepcidin (ng/ml)	8.86 (0.11, 103.78)	8.70 (0.17, 94.64)	9.00 (0.11, 103.78)	0.780
TSAT (%)	20.73 (2.84, 75.72)	20.07 (2.84, 57.51)	21.42 (4.31, 75.72)	0.114
Transferrin (g/l)	2.75 (0.01, 4.77)	2.74 (0.00, 4.21)	2.77 (0.00, 4.77)	0.009
TIBC (umol/l)	60.4 (1.4, 129.3)	60.0 (20.5, 129.3)	60.8 (1.4, 123.0)	0.028
UIBC (umol/l)	47.3 (0.8, 120.8)	47.3 (11.8, 120.8)	47.2 (0.8, 113.4)	0.642
Ferritin (ug/l)	26.9 (0.10 (166.8)	27.7 (0.2, 161.7)	25.3 (0.1, 166.8)	0.063
sTfR (mg/l)	4.93 (0.70, 19.77)	4.84 (0.79, 14.11)	4.41 (0.00, 19.77)	0.083
CRP (mg/l)	1.19 (0.0, 40.26)	1.11 (0.00, 40.26)	1.23 (0.00, 32.24)	0.083

Data are presented in median (ranges), except gender.

*CRP* C-reactive protein, *Hb* haemoglobin, *MCV* mean corpuscular volume, *MCH* mean corpuscular haemoglobin, *MCHC* mean corpuscular Hb concentration, *RDW* red cell distribution width, *RBC* red blood cells, *sTfR* soluble transferrin receptor, *TIBC* total iron-binding capacity, *UIBC* unsaturated iron-binding capacity, *TSAT* transferrin saturation.

### *TMPRSS6* variants

**Figure 1:**
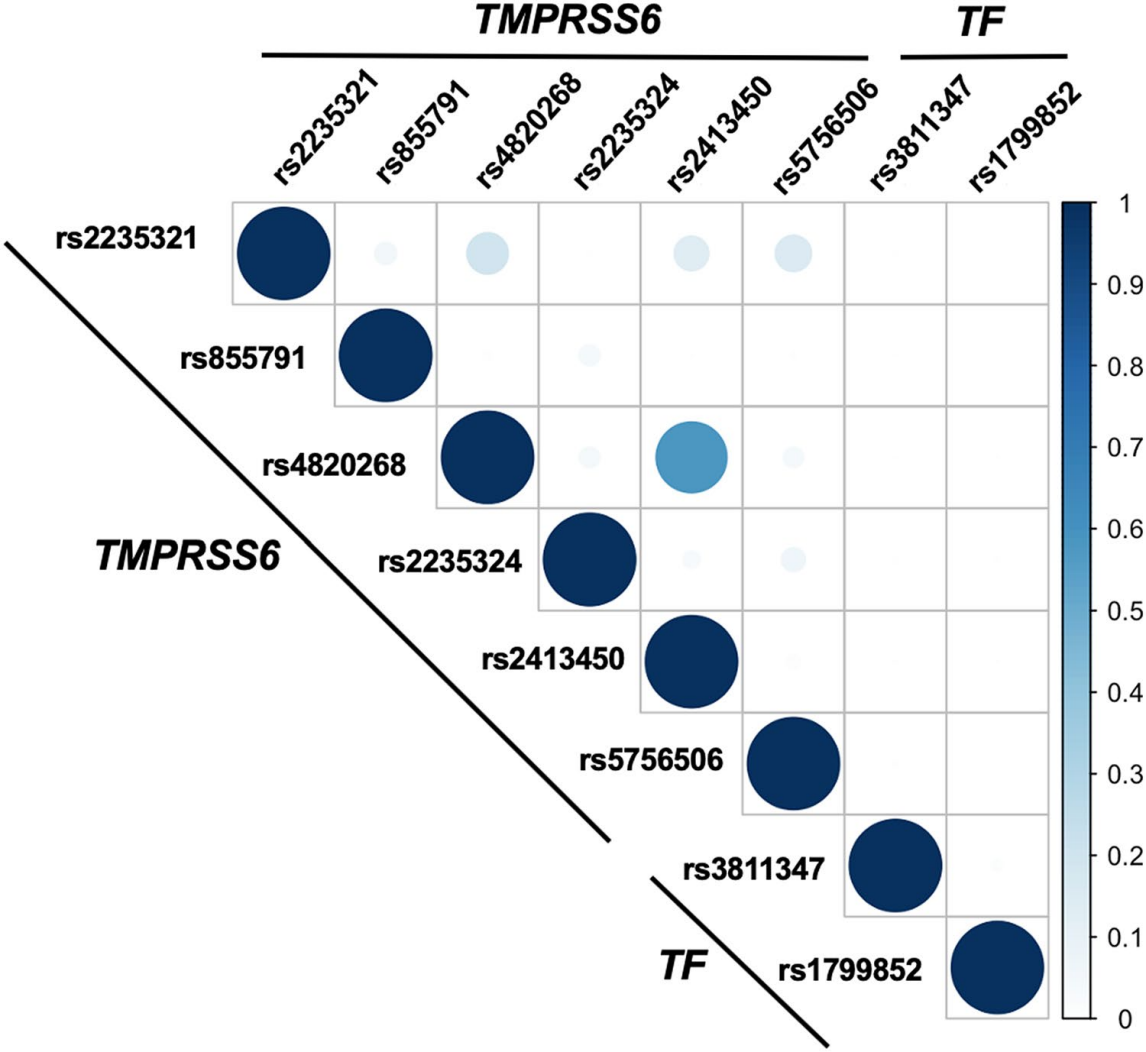
Linkage disequilibrium analysis between SNPs investigated in this study.

All the SNPs investigated were in Hardy–Weinberg Equilibrium. Also, all were in low linkage disequilibrium (LD) in this study population, except rs4820268 and rs2413450 which have *r*^2^ = 0.7 (Fig. 1). Among the SNPs we investigated, *TMPRSS6* rs2235324 had the highest minor allele frequency (MAF) in our study population (45%), and *TMPRSS6* rs855791 and *TF* rs1799852 had the lowest MAF (7% each)^24^.

**Figure 2:**
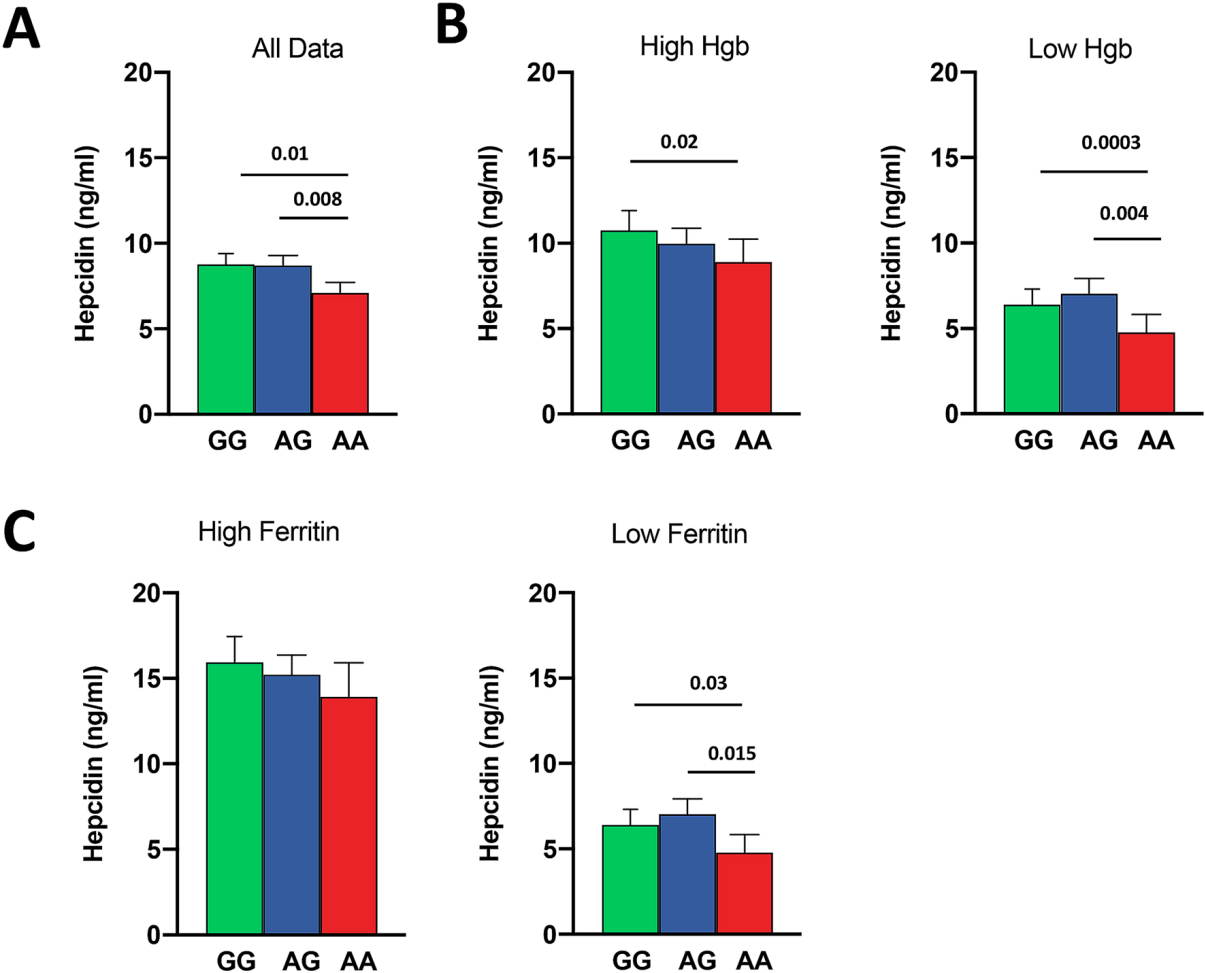
The effects of *TMPRSS6* rs2235321 on plasma hepcidin levels. (**A**) All data (GG n = 416, GA n = 586, AA n = 262). ANOVA P for trend = 0.004. (**B**) Sample divided into high and low Hb (< 11.5 g/dl). High Hb (GG n = 245, GA n = 344, AA n = 162). ANOVA P for trend = 0.02. Low Hb (GG n = 171, GA n = 242, AA n = 100). ANOVA P for trend = 0.0002. (**C**) Sample divided above and below median ferritin (< 26 ng/ml). High ferritin (GG n = 247, GA n = 313, AA n = 155). ANOVA P for trend = 0.0004. Low ferritin (GG n = 169, GA n = 273, AA n = 107). ANOVA P for trend = NS.

There was no detectable influence of sex on any associations, so sex was discarded from the models. None of the *TMPRSS6* SNPs studied showed any association with any of the iron status markers (ferritin, serum iron, transferrin, TSAT, sTfR, TIBC or UIBC) or haematological variables (Hb, MCV, MCH, MCHC, RDW, RBC or Hct) or with CRP. However, hepcidin levels varied significantly by rs2235321 genotype (Fig. 2A) with lower hepcidin in the AA homozygotes, 19% than GG carriers (F ratio 3.70, P = 0.014). Note that Bonferroni correction for having analysed 6 SNPs would render the rs2235321 of marginal significance. These trends were stronger in subjects with lower Hb (Fig. 2B) and lower ferritin levels (Figs. 2C). The other SNPs had no detectable influence on hepcidin.

**Figure 3:**
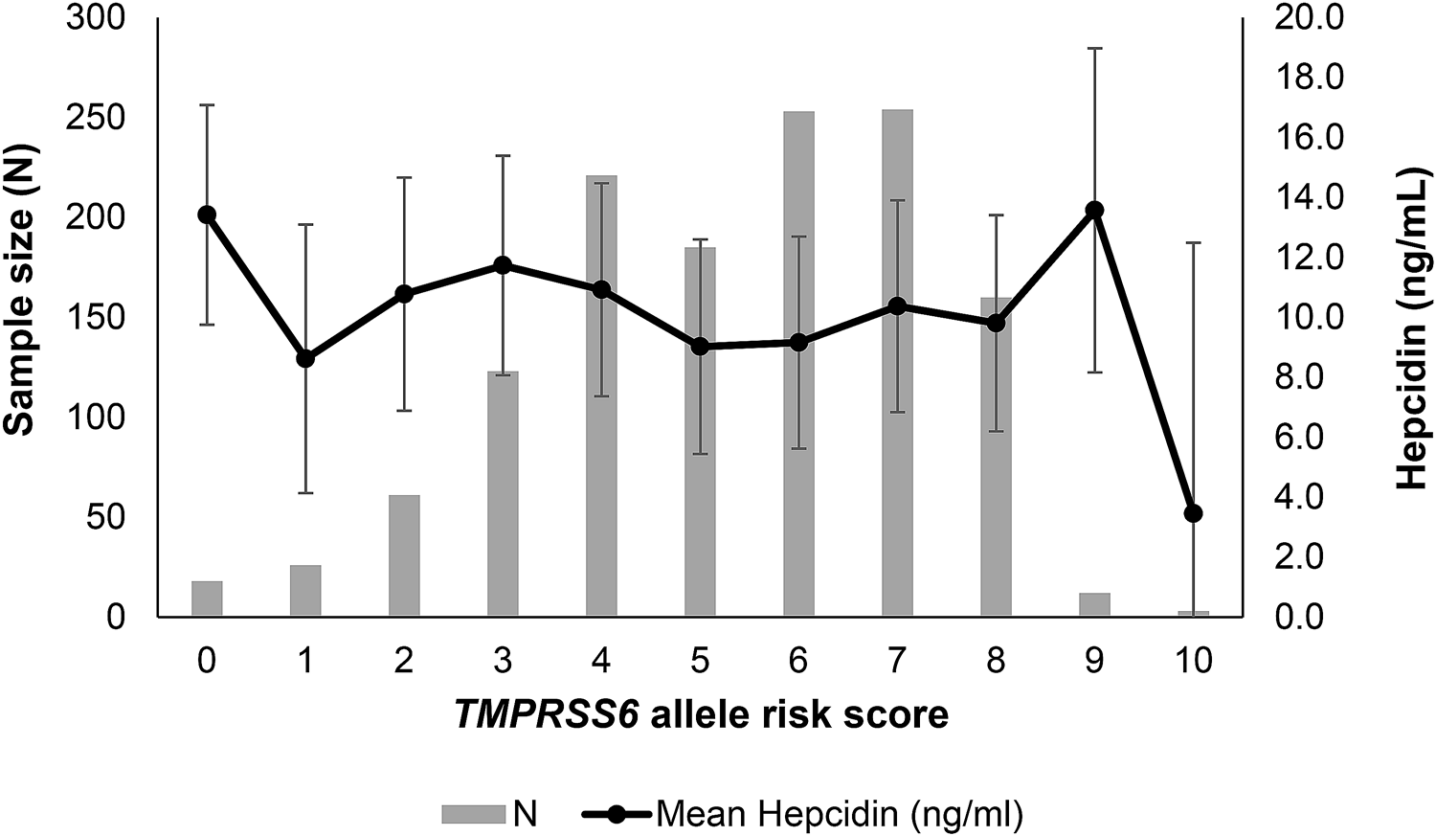
Influence of *TMPRSS6* allele risk score on plasma hepcidin. Error bars = standard errors (SE).

Despite the lack of significant association with 5 of the 6 SNPs, we investigated whether allele risk score (ARS) was a significant predictor of hepcidin by using published data on the direction of association to allocate a score of 0, 1 or 2 to allele combinations for each SNP and summing the scores across all SNPs (Fig. 3). ANOVA across all ARS revealed no association with plasma hepcidin (F-ratio = 1.01, *P* = 0.458).

### *TF* variants

**Figure 4:**
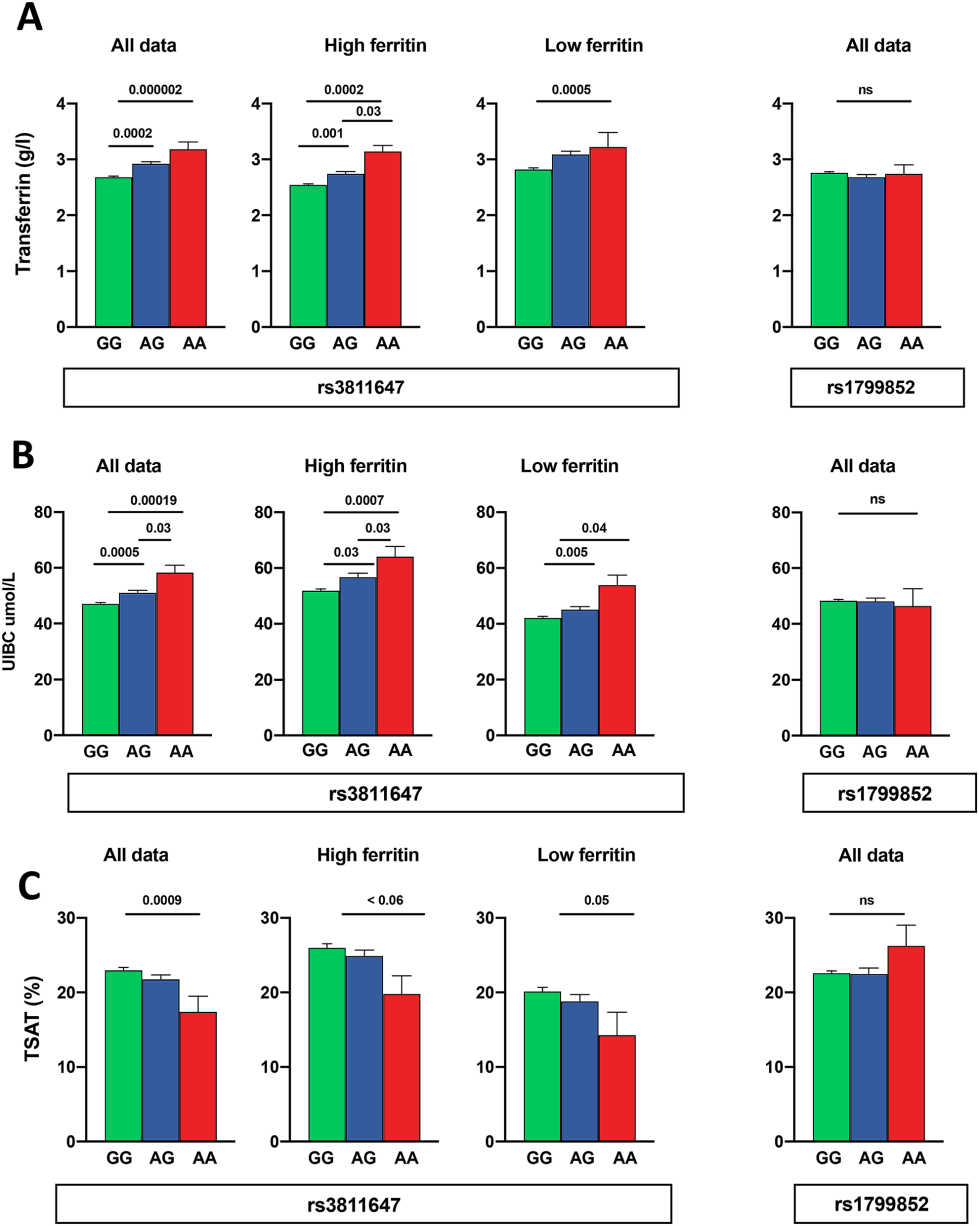
The effects of *TF* rs3811647 and rs1799852 on plasma iron binding capacity and TSAT. (**A**) Transferrin rs3811647 All data (GG n = 720, GA n = 215, AA n = 24). ANOVA P for trend < 0.0001. Sample divided above and below median ferritin (< 26.9 ng/ml). High ferritin (GG n = 360, GA n = 108, AA n = 15). ANOVA P for trend < 0.0001. Low ferritin (GG n = 360, GA n = 107, AA n = 11). ANOVA P for trend ≤ 0.0001. rs1799852 All data (GG n = 839, GA n = 116, AA n = 4). ANOVA P for trend = NS. (**B**) UIBC rs3811647 All data (GG n = 985, GA n = 301, AA n = 28). ANOVA P for trend < 0.0001. Sample divided above and below median ferritin (< 26 ng/ml). High ferritin (GG n = 474, GA n = 147, AA n = 16). ANOVA P for trend < 0.0001. Low ferritin (GG n = 511, GA n = 154, AA n = 12). ANOVA P for trend < 0.0001. rs1799852 All data (GG n = 1139, GA n = 116, AA n = 9). ANOVA P for trend = NS. (**C**) TSAT rs3811647 All data (GG n = 720, GA n = 215, AA n = 24). ANOVA P for trend < 0.0001. Sample divided above and below median ferritin (< 26 ng/ml). High ferritin (GG n = 360, GA n = 108, AA n = 15).

The two *TF* SNPs were also tested in combination and individually. Rs1799852 showed no association with any outcomes. However, rs3811647 was strongly associated with transferrin levels (Fig. 4A) with or without the inclusion of sex and age as co-variables (F ratio 16.0, P < 0.0001). There was an apparent allele dose effect with AA homozygotes having 21% higher transferrin than GG. TIBC (partially computed from transferrin) was similarly affected with a 16% higher value for the AA genotype (F ratio 14.0, P < 0.0001).

UIBC, which is directly measured rather than computed, showed the same pattern (Fig. 4B) with 24% higher values in individuals carrying AA (F ratio 12.8, P < 0.0001). Serum iron was not significantly associated with the rs3811647 genotype. So, on account of the raised TIBC and transferrin, TSAT was lower in the AA group (by 25%, with a single allele effect of 12.5%) (F ratio 4.3, P < 0.0001) (Fig. 4C). The transferrin, UIBC and TSAT associations were robust when we separated the subjects into above and below the median ferritin value (Fig. 4A–C). None of the other iron markers was affected nor was there any influence on any of the haematological markers, hepcidin or CRP.

## Discussion

Based upon prior GWAS studies, pathway analysis, availability on the Illumina Exome Array, and having a high minor allele frequency in our Gambian population we studied the effect of six candidate SNPs in *TMPRSS6* and two in *TF* on multiple indices of iron and haematological status in 1316 individuals from 1 to 87 years age, from the Keneba Biobank at the MRCG @ LSHTM, in the Gambia. We found weak evidence that one *TMPRSS6* SNP (rs2235321) had lower hepcidin levels in the variant (AA) homozygotes with an indication of an allele dose effect. One *TF* variant (rs3811647) showed increased transferrin, TIBC and UIBC levels in the AA homozygotes and lower levels of TSAT.

In this population, none of the other variants was significantly associated with any of the iron, haematological, hepcidin or inflammation markers. Applying an allele risk score approach to the 6 *TMPRSS6* variants also yielded no detectable association with any outcomes. Most previous research on the effects of our candidate SNPs were conducted in non-African populations, and there is no prior data on West Africans. The associations we observed differ from results obtained in other populations. *TMPRSS6* rs855791 is a non-synonymous SNP that has been widely reported to influence iron parameters and to be associated with the risk of IDA in Europeans^14,19^ and Asians^12,26^. We found no such associations between rs855791 and iron status biomarkers.

The *TMPRSS6* rs2235321 is a synonymous variant which has been reported to associate with benign microcytic anaemia^27^. We did not find any other reported associations. Our data confirm a null effect on hepcidin even in a population with high levels of anaemia and low iron status. A meta-analysis of GWAS on the genetic determinants of hepcidin did not identify any *TMPRSS6* SNP that is significantly associated with hepcidin concentration^13^. It is important to notice that our candidate gene approach demonstrated a relatively small effect of *TMPRSS6* rs2235321 on hepcidin (single allele effect of 9.5%), and this needs to be considered when designing GWAS.

*TF* rs3811647 is an intron variant on the transferrin gene with extensive prior evidence for functional effects. In discovery and replication GWAS analyses of cohorts from Italy and the USA, Pichler et al.^28^ confirmed the association between rs3811647 and transferrin levels. In a subsequent GWAS analysis, Mclaren et al. showed that *TF* rs3811647 is associated with serum TIBC^19^. Also, Blanco-Rojo et al.^21^ demonstrated that rs3811647 influenced transferrin gene expression in liver. Previously, Benyamin et al. showed that three variants in *TF* (rs3811647, rs1799852 and rs2280673) plus the *HFE* C282Y mutation explained ∼40% of genetic variation in serum transferrin (p = 7.8 × 10^−25^)^17^. Our data are suggestive of an allele dose–response relationship with a single allele effect of 9.5% for transferrin levels (higher with the A variant) and a reverse effect of about 12.5% for TSAT.

Other investigators have reported associations between the *TF* rs1799852 and iron status. In a study of female black South Africans, Gichohi et al. reported that heterozygotes at *TF* rs1799852 (AG) had lower iron status (low serum ferritin and body iron, and higher sTfR concentrations) than the homozygotes (AA)^23^. This suggested that rs1799852 AA might be protective against low iron status. Similarly, Benyamin et al. reported that the *TF* rs1799852 was associated with lower transferrin concentration and the risk of haemochromatosis^29^. Furthermore, Blanco-Rojo et al.^18^ reported that the *TF* rs1799852 A allele was associated with low serum transferrin concentration, and it compensated for the effect of rs3811647 A allele on the risk of IDA. The authors further suggested that carrying *TF* rs3811467 G allele simultaneously with rs1799852 A allele and *HFE* C282Y and H63D might be protective against low iron status as they increase the susceptibility to iron overload^18^. In the present study, we could not include the *HFE* C282Y and H63D variants, because their MAF in Gambians is extremely low (0.4% and 0% respectively)^30^. Our failure to replicate the prior findings for rs1799852 may be ascribed to the fact that we had only nine individuals homozygous for the A allele. However, in contrast to the South African data^23^, we also found no evidence for differences in any of the iron markers between rs1799852 GG (n = 854) and AG (n = 117).

This study has strengths and weaknesses. We used highly standardised laboratory assays to measure seven markers of iron status, seven haematological traits, plus hepcidin and CRP. The sample size was large in the context of candidate gene studies, and we spanned the age range 1–87 years (with appropriate adjustment for age and sex in the analyses). The population generally has marginal iron status and high levels of anaemia which might better expose underlying genetic effects. This may suggest that the impact of underlying genetic effects on iron status may be amplified in individuals with low iron status.

One of the limitations of the study is that we had only six *TMPRSS6* and two *TF* SNPs available on the exome chip. By definition, these SNPs had been curated onto the chip because of prior evidence of functionality. Another limitation is that for some of the SNPs (notably rs1799852 discussed above) we had very few individuals homozygous for the variant allele. In conducting this study, our initial objective was to explore whether common genetic variants in iron regulatory pathways might have a significant influence on the risk of iron deficiency and iron-deficiency anaemia in African populations. Despite selecting the variants with some strong prior evidence of functionality, our data did not reveal significant genetic effects that on the risk of anaemia to support stratified medicine approach in African population. Even where we observed associations (effects of *TMPRSS6* rs2235321 on plasma hepcidin, and *TF* rs3811647 on iron transporting capacity and TSAT), the single allele effect sizes approximated 10%, and there were few people homozygous for the variant allele. Furthermore, there may be inherent compensatory mechanisms because none of the variants had any effect on other markers of iron or haematological status.

In this study, we identified a modest association between *TMPRSS6* rs2235321 and hepcidin and replicated the previous findings on the effects of *TF* rs3811647 on serum transferrin. However, the overall population attributable risk conferred by these known genetic factors is negligible. As these variants were mainly studied in European and Asian populations, it is possible that other genetic variants in these genes will be more informative for iron studies in African populations, and this needs to be addressed to develop genetically stratified approaches to prevention or treatment of iron deficiency anemia.

## Methods

### Study populations and sample collection

This study utilised a cohort of healthy individuals, enrolled in the Keneba Biobank at MRCG@LSHTM^31^. Based on the availability of genotype data, we studied 1316 individuals aged 1–87 years (54.2% females). Each participant was interviewed and had a basic health examination, and those with significant health conditions excluded. After an overnight fast, a venous blood sample was collected in EDTA and lithium heparin tubes. DNA was extracted from cell pellets using standard procedures and stored at − 70 °C. Plasma samples from lithium heparin anticoagulant were stored at − 70 °C freezers until analysis.

### Haematology and iron biomarker measurements

Full blood count (FBC) was performed within 4 h of sample collection using a Medonic M-Series automated haematology analyser (Boule Medical, Sweden) and results analysed for haemoglobin (Hb), mean corpuscular volume (MCV), mean corpuscular haemoglobin (MCH), mean corpuscular Hb concentration (MCHC), red cell distribution width (RDW), red blood cell number (RBC) and haematocrit (Hct). Iron biomarkers [serum iron, transferrin, ferritin, unsaturated iron-binding capacity (UIBC), soluble transferrin receptor (sTfR) and the inflammation marker C-reactive protein (CRP)] were measured using an automated biochemistry analyser (COBAS Integra 400 Plus, Roche Diagnostics). Total iron-binding capacity (TIBC) and transferrin saturation (TSAT) were calculated from UIBC and plasma iron (TIBC = plasma iron + UIBC) and TSAT = [plasma iron/TIBC] × 100). Plasma hepcidin was quantified using competitive ELISA (Bachem Hepcidin-25; Penninsula Laboratories International).

### Genotyping

This study population was genotyped using the Illumina Infinium 240 K Human Exome Beadchip (v1.0 and v1.1), as previously described^24^, in which 848 SNPs were genotyped. Genotype calling was performed using data-driven clustering (Genome Studio, Illumina, CA, USA). The *TMPRSS6* rs2235321, rs855791, rs4820268, rs2235324, rs2413450 and rs5756506, and *TF* rs3811647 and rs1799852 were selected for inclusion in this study based on their previously published association with iron status.

### Genotype combinations and allele risk scores

For both the *TMPRSS6* and *TF* SNPs, we generated genotype combinations and allele risk scores (ARS) by summing up the genotypes and the number of risk alleles respectively, from all the SNPs an individual carried. Risk alleles were defined as the alleles that are previously reported to be associated with iron-lowering at each SNP. For each SNP, genotypes were assigned 0, 1 or 2, with risk alleles assigned 1 and the alternate allele assigned 0. Thus, homozygous for the risk allele scored 2 and homozygous for non-risk alleles were scored 0, Table S1 (*TMPRSS6* SNPs) and Table S2 (*TF* SNPs).

A total of 94 genotype combinations from the six *TMPRSS6* SNPs were found in our population (Table S3). We investigated the effects of individual *TMPRSS6* and *TF* SNPs on all the iron biomarkers and haematology phenotypes. In addition, based on the functional role of *TMPRSS6* on hepcidin regulation, and its effects on iron status, we investigated the effects of *TMPRSS6* ARS on hepcidin (Table S4). Similarly, we assessed the effects of *TF* SNPs’ genotype combinations and *TF* ARS on transferrin level.

### Statistical analysis

The effects of genetic variants (genotypes of single SNPs or combinations of multiple SNPs) on iron biomarkers were determined by linear modelling with iron and haematological traits as response variables and genotype as dependent variables. Age, sex, inflammation (CRP) were added as covariates where indicated. We tested the effects each SNP individually and in combinations on iron biomarkers. Hepcidin, ferritin and CRP were log transformed. We added log ferritin as a covariate when analysing the effects of genotype on plasma hepcidin because *TMPRSS6* modulates the interaction between iron status and hepcidin gene expression. Furthermore, we stratified the study population based on haemoglobin and ferritin levels and determined the effects of genotype on each sub-population. For each sub-population, we used analysis of variance (ANOVA) to determine the effects of individual SNPs on iron biomarkers. Bonferroni correction was applied to account for multiple testing. The statistical analyses were conducted using R statistical software^32^ and DataDesk Version 7.0.2 (Data Description Inc, Ithaca).

### Ethics

The study was approved by the MRCG@LSHTM/Gambia Government Ethics Committee (SCC1185). A written informed consent was obtained from each study participant at the age of 18 years and above. For participants under the age of 18 years, a written informed consent was obtained from a parent and/or a legal guardian. All experiments were conducted in accordance with the MRCG at LSHTM and Gambia Government Ethics Committee guidelines and in accordance with the guidelines of the Helsinki Declaration.

### Consent for publication

The consent for publication has been obtained from all the participants.

## Supplementary Information

Supplementary Information.

## Data Availability

The datasets generated and/or analysed during the current study are available upon written request sent to the MRCG at LSHTM/Gambia Government Joint Ethics Committee.

## Abbreviations

GWAS: Genome-wide association studies
SNPs: Single nucleotide polymorphisms
IRIDA: Iron-refractory iron deficiency anaemia
*TMPRSS6*: Transmembrane protease serine 6
*TF*: Transferrin
HFE: Haemochromatosis factor
ARS: Allele risk score
FBC: Full blood count
RBC: Red blood cells
TSAT: Transferrin saturation
sTfR: Soluble transferrin receptor
UIBC: Unsaturated iron binding capacity
TIBC: Total iron binding capacity
CRP: C-reactive protein
LD: Linkage disequilibrium
EDTA: Ethylenedimethyltetraacetic acid
DNA: Deoxyribonucleic acid
MCV: Mean corpuscular volume
HCT: Haematocrit
MCH: Mean corpuscular haemoglobin
MCHC: Mean corpuscular haemoglobin concentration
ELISA: Enzyme-linked immunosorbent assay
MRCG at LSHTM: Medical Research Council Unit the Gambia at London School of Hygiene & Tropical Medicine
NFKBIL1: Nuclear factor kB inhibitor-like protein 1
