# Supplementary material for: Association of common TMPRSS6 and TF gene variants with hepcidin and iron status in healthy rural Gambians
Source: Sci Rep. 2021 Apr 13;11:8075. doi: 10.1038/s41598-021-87565-5 (PMC8044158; doi:10.1038/s41598-021-87565-5)
Supplement: Supplementary file 1 — Supplementary Information. [file 41598_2021_87565_MOESM1_ESM.pdf]

Supplemental Data

Association of common *TMPRSS6 and TF* gene variants with hepcidin and iron status in healthy rural Gambians.

Momodou W. Jallow, Susana Campino, Andrew M. Prentice and Carla Cerami

Supplemental information

Supplemental Table S1. Allele configuration of the allele risk score from the 6 *TMPRSS6* SNPs

SNP	rs2235321	rs855791	rs4820268	rs2235324	rs2413450	rs5756506	Total Risk score
major/minor	G/A	G/A	A/G	A/G	G/A	G/C	
Risk allele*	**A**	**A**	**A**	**G**	**G**	**C**	
normal allele	G	G	G	A	A	G	
Risk genotype (ARS)	**A/A (2)**	**A/A (2)**	**A/A (2)**	**G/G (2)**	**G/G (2)**	**C/C (2)**	**12**
normal group	G/G (2)	G/G (0)	G/G (0)	A/A (0)	A/A (0)	G/G (0)	0

ARS, allele risk score

* Allele linked to low iron status from the previously-published studies
Bolded alleles are those associated with the risk of low iron status.

Supplemental Table S2. Details of the allele configuration for the allele risk score from the 2 *TF* SNPs

SNP	rs1799852	rs3811647	Genotype combination	ARS	N
major/minor	G/A	G/A			
Risk allele*	**G**	**A**			
WT/WT	**GG**	GG	**GG**/GG	2	825
Het/Het	A**G**	**A**G	A**G**/**A**G	2	10
Homo/Homo	AA	**AA**	AA/AA	2	0
WT/Homo	**GG**	**AA**	GG/AA	4	27
Homo/WT	AA	GG	AA/GG	0	9
Het/WT	A**G**	GG	AG/GG	1	154
WT/Het	**GG**	AG	GG/**A**G	3	290
Het/Homo	A**G**	AA	AG/**AA**	3	1
Homo/Het	AA	AG	AA/AG	1	0

* Allele linked to low iron status from previously-published studies; bolded letters indicates risk alleles

Supplemental Table S3. A list of all the 94 genotype combinations generated from the 6 *TMPRSS6* SNPs

Genotype group	N	*TMPRSS6* SNP ARS
**GG/GG/GG/AA/AA/GG**	18	0
GG/GG/GG/AA/AG/GG	16	1
GG/GG/GG/GA/AA/GG	10	1
GG/GG/GA/AA/AG/GG	26	2
GG/GG/GG/GA/AG/GG	20	2
AG/GG/GG/AA/AG/GG	4	2
AG/GG/GG/GA/AA/GG	4	2
GG/AG/GG/AA/AA/CG	3	2
GG/GG/GG/GG/AA/GG	3	2
GG/GG/GG/GG/AA/GG	2	2
GG/GG/GG/AA/GG/GG	1	2
GG/GG/GG/GA/AA/CG	1	2
AG/GG/GA/AA/AG/GG	72	3
GG/GG/GA/AA/AG/CG	16	3
GG/GG/GA/AA/GG/GG	16	3
GG/GG/GA/GA/AG/GG	12	3
AA/GG/GG/AA/AG/GG	1	3
AG/GG/GA/NA/AG/NA	1	3
AG/GG/GG/GA/AG/GG	1	3
GG/AG/GA/AA/AG/GG	1	3
GG/AG/GG/GA/AA/CG	1	3
AG/GG/GA/GA/AG/GG	81	4
GG/GG/GA/GA/AG/CG	52	4
AG/GG/GA/AA/GG/GG	29	4
GG/AG/GA/GA/AG/GG	20	4
GG/GG/AA/AA/GG/GG	14	4
GG/GG/GA/GA/GG/GG	9	4
GG/GG/GA/AA/GG/CG	6	4
AG/AG/GG/AA/AG/CG	2	4
AG/GG/GG/GA/GG/GG	2	4
GG/GG/GA/GG/AG/GG	2	4
GG/GG/GG/GG/GG/GG	2	4
AA/GG/GA/AA/AG/GG	1	4
GG/GG/GG/GG/AG/CG	1	4

Supplemental Table S3 cont.

Genotype group	N	*TMPRSS6* SNP ARS
AG/GG/AA/AA/GG/GG	42	5
AG/GG/GA/GA/GG/GG	42	5
GG/GG/GA/GA/GG/CG	25	5
AG/GG/GA/GG/AG/GG	16	5
GG/GG/GA/GG/AG/CG	8	5
GG/GG/AA/GA/GG/GG	7	5
AA/GG/GA/AA/GG/GG	5	5
AG/AG/GA/AA/AG/CG	5	5
GG/AG/GA/GA/AG/CG	5	5
GG/GG/AA/AA/GG/CG	5	5
GG/AG/GA/GA/GG/GG	4	5
AA/GG/GA/GA/AG/GG	3	5
AG/GG/GA/AA/GG/CG	3	5
GG/GG/GA/GG/GG/GG	2	5
AG/AG/GA/GA/AG/GG	1	5
AG/GG/GA/GA/AG/CG	1	5
GG/AG/AA/AA/GG/GG	1	5
GG/AG/GA/AA/AG/CC	1	5
NA/GG/AA/GA/GG/GG	1	5
AA/GG/AA/AA/GG/GG	75	6
AG/GG/AA/GA/GG/GG	56	6
AG/GG/AA/AA/GG/CG	24	6
GG/GG/AA/GA/GG/CG	20	6
GG/AG/AA/GA/GG/GG	19	6
AG/GG/GA/GG/GG/GG	16	6
AA/GG/GA/GA/GG/GG	12	6
GG/GG/GA/GG/GG/CG	7	6
GG/AG/GA/GG/GG/GG	5	6
AA/GG/GA/GG/AG/GG	4	6
AG/AG/GA/GA/AG/CG	3	6
AG/GG/GA/GA/GG/CG	2	6
GG/AG/GA/GA/GG/CG	2	6
GG/GG/AA/AA/GG/CC	2	6
AG/AG/GA/GA/GG/GG	1	6
AG/GG/GA/GG/AG/CG	1	6

Supplemental Table S3 cont.

Genotype group	N	*TMPRSS6* SNP ARS
GG/AG/GA/GA/AG/CC	1	6
GG/GG/AA/GG/GG/GG	1	6
NA/GG/AA/GA/GG/CG	1	6
AA/GG/AA/GA/GG/GG	123	7
AG/GG/AA/GA/GG/CG	62	7
AG/AG/AA/GA/GG/GG	32	7
AG/GG/AA/GG/GG/GG	19	7
GG/GG/AA/GA/GG/CC	8	7
GG/GG/AA/GG/GG/CG	6	7
GG/AG/AA/GA/GG/CG	4	7
GG/AG/AA/GG/GG/GG	2	7
AG/GG/AA/GG/GG/CG	50	8
AA/GG/AA/GG/GG/GG	46	8
AG/AG/AA/GG/GG/GG	30	8
GG/GG/AA/GG/GG/CC	14	8
GG/AG/AA/GG/GG/CG	13	8
AG/AG/AA/GA/GG/CG	6	8
GG/AG/GA/GG/AG/GG	6	8
GG/AA/AA/GG/GG/GG	1	8
GG/AG/AA/GG/GG/CC	6	9
AG/AG/AA/GG/GG/CG	2	9
AG/GG/AA/GG/GG/CC	2	9
AA/GG/AA/GG/GG/CG	1	9
GG/AA/AA/GG/GG/CG	1	9
GG/AA/AA/GG/GG/CC	3	10

Abbreviations: ARS, allele risk score
Legend: NA, genotypes that were not available for that particular SNP in the combination.
TMPRSS6 SNP ARS, number of iron-lowering alleles within a genotyoe combination

Supplemental Table S4. The effects of *TMPRSS6* allele risk score on hepcidin, controlling for age, sex and CRP

TMPRSS6 ARS*	N	Mean Hepcidin (ng/ml)	Std. error	Beta	*P*-value
0	18	13.4	3.66	Reference group	
1	26	8.6	4.48	-4.80	0.285
2	61	10.8	3.89	-2.64	0.498
3	123	11.7	3.66	-1.68	0.645
4	221	10.9	3.55	-2.49	0.483
5	185	9.0	3.58	-4.39	0.220
6	253	9.2	3.54	-4.26	0.229
7	254	10.4	3.54	-3.04	0.390
8	160	9.8	3.61	-3.61	0.317
9	12	13.6	5.41	0.16	0.977
10	3	3.5	9.04	-9.96	0.270

* Number of risk alleles based on **Table S1**
